# Molecular Dynamics Simulation of Effect of Carbon Nanotube Diameter on Properties of Crosslinked Epichlorohydrin Rubbers

**DOI:** 10.3390/polym16172419

**Published:** 2024-08-26

**Authors:** Zepeng Wang, Xinyan Li, Liangchen Yu, Junping Song

**Affiliations:** 1 College of Electromechanical Engineering, Qingdao University of Science and Technology, Qingdao 266061, China; lixinyan4556203@163.com (X.L.); 4022030135@mails.qust.edu.cn (L.Y.); 2 Sino-German Science College, Qingdao University of Science and Technology, Qingdao 266061, China; 02165@qust.edu.cn

**Keywords:** crosslinking, epichlorohydrin rubber, carbon nanotubes, mechanical properties, molecular dynamics simulation

## Abstract

Molecular dynamics simulation (MD) technology can be used to simulate and study the physicochemical properties of polymer materials on the basis of material data obtained in traditional experiments. In this study, we use MD to construct models of crosslinked carbon nanotubes/epichlorohydrin rubber composites with different carbon nanotube diameters and study the effect of CNT tube diameter on crosslinked ECO. The results show that with the increase in CNT tube diameter, the contact area between the CNTs and rubber matrix increases, the interaction force is enhanced, the free volume fraction of rubber matrix decreases by 21.36%, the glass transition temperature increases by 6.5%, the mean square displacement decreases, the radius of gyration decreases by 3.18 Å, and the radial distribution function of the C-atom group and the H-atom group in the system gradually decreases. The binding energy between the two is elevated, which in turn verifies the enhancement of the interaction force.

## 1. Introduction

Epichlorohydrin rubber, as an important member of special rubber, currently has four major manufacturers in the world, which are Hebei Lixing Special Rubber Co., Ltd.; Wuhan Youji Industrial Co., Ltd.; Japan Ruiweng Company; and Japan Dacao Company [1]. However, an American manufacturing company, Goodrich and Hercules, in the 1970s achieved the world’s earliest industrial production of epichlorohydrin rubber, and then China, in the late 1970s, began to research and prepare, and then began mass production throughout the country. With the excellent properties of epichlorohydrin rubber being widely noticed, its quality and quantity have been continuously improved, and it has been applied in many fields such as military, aviation, machinery, electronics, and automotive [2]. Although the research of China’s epichlorohydrin rubber has already been at the top level in the world, domestic epichlorohydrin rubber still has a certain gap in terms of the production scale, quality and performance between the products of Japan and the United States.

Most of the current research on epichlorohydrin rubber has been conducted using macro-experiments; the use of molecular dynamics at the microscopic level of study has been less. Zhao Hongyu et al. [3] explored the effect of carbon black types and dosages of epichlorohydrin rubber, using N330, N550, and N774 on the dimer copolymerisation of epichlorohydrin rubber mechanical properties, electrical properties, and other aspects. The study showed that carbon black N330 and N550 have the same effect on the torque of epichlorohydrin rubber, and both of their effects are greater than the effect of carbon black N774 on the torque of epichlorohydrin rubber. The hardness of the colloid increased with the amount of reinforcing material, with carbon black N774 having the least effect on the hardness of the colloid. The effects of carbon blacks N550 and N774 on the tensile strength and elongation at break of the colloid were less than those of carbon black N330. Zhipeng Y et al. [4] investigated the effect of black liquor–montmorillonite (BL-Mnt) on the mechanical and thermal properties of binary copolymerised chlorinated ether rubbers by applying x-ray diffraction, transmission electron microscopy, and thermogravimetric analysis. The results showed that the fillers were well dispersed in the composites, and the best tensile strength of 14.0 MPa and elongation at break of 457% were obtained when 50% black liquor–montmorillonite was added to ECO, and the 100% modulus was the largest at 7.2 MPa when the filler amount of black liquor–montmorillonite was 90%. Timofeeva E et al. [5] investigated the effect of multi-walled carbon nanotubes on the properties of epichlorohydrin rubber by adding them to epichlorohydrin rubber. Their research showed that the strength of rubber increases with the increase in the content of multi-walled carbon nanotubes, but the high concentration of multi-walled carbon nanotubes will significantly reduce the relative elongation of rubber. Among the many performance changes, the wear resistance of rubber is the most affected by the addition of multi-walled carbon nanotubes, which can be increased by 30%.

## 2. Model Construction

### 2.1. Crosslinking ECO Model Construction

**Figure 1:**
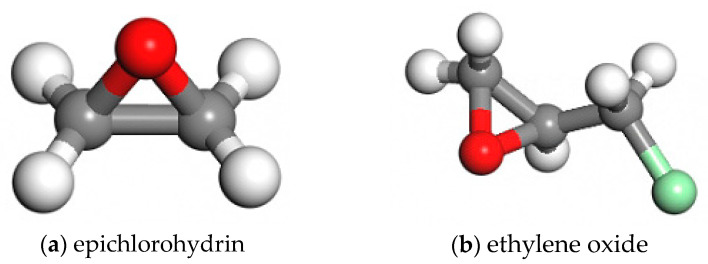
Structure diagrams of ECH and EO.

**Figure 2:**
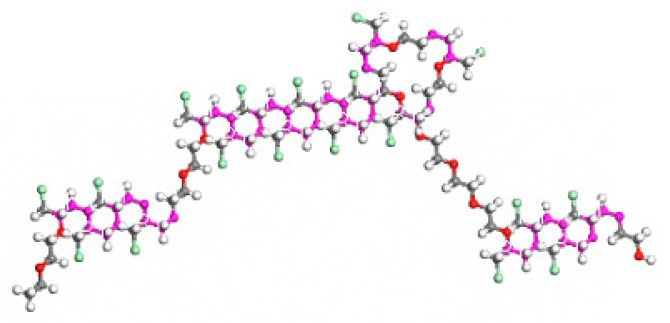
Molecular chain of ECO.

The dimer copolymerised epichlorohydrin rubber is a copolymer of ethylene oxide (EO) and epichlorohydrin (ECH) [6]. The monomer models of ethylene oxide and epichlorohydrin were first constructed with Materials Studio 2019 [7], and their structures are shown in Figure 1. Then, a molecular chain of ECO rubber with a polymerisation degree of 30 was constructed from ethylene oxide and epichlorohydrin at a ratio of 4:6 using Build Polymers of the Build module, and the structure is shown in Figure 2.

**Figure 3:**
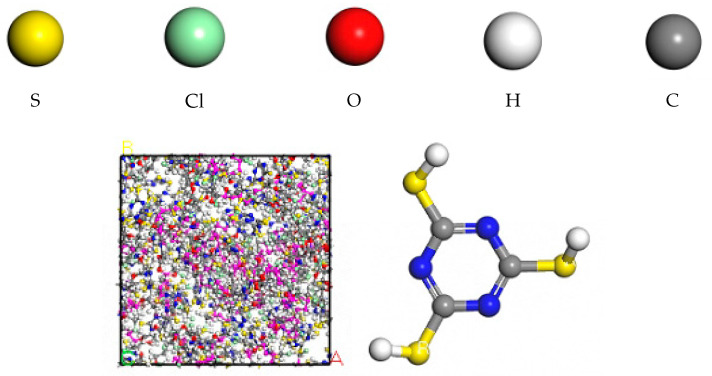
ECO initial model and structure diagram of TCY.

**Figure 4:**
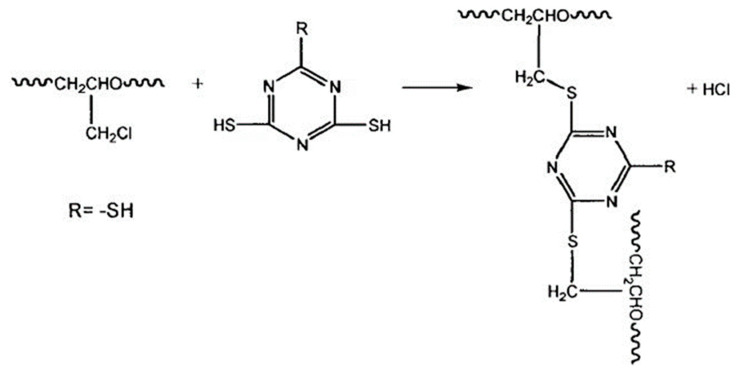
Vulcanisation mechanism of epichlorohydrin rubber.

The constructed ECO molecular chains with the thiolator triazine thiol (TCY) were randomly populated into the cubic lattice according to Monte Carlo’s rule with the Construction function in the Amorphous Cell module until the density of the cubic lattice reached 1.2 g/cm^3^. The model is shown in Figure 3. The principle of vulcanisation is shown in Figure 4.

**Figure 5:**
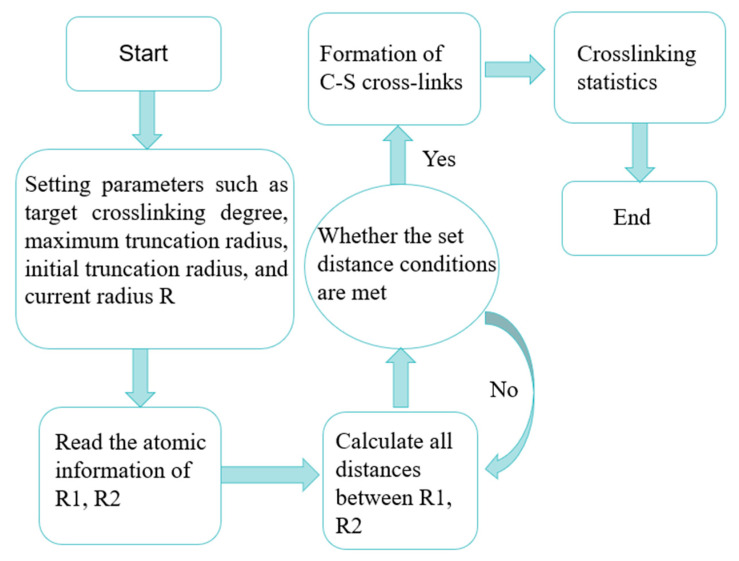
Crosslinking flowchart.

After the model was established, all the carbon atoms connected by chlorine atoms in the ECO rubber model were renamed as R1, and any two sulphur atoms in the TCY were named as R2, and then the model was vulcanised and crosslinked with an epoxy crosslinking script to form a carbon–sulphur bond (-C-S-C-) in the model, with the following process: (1) Read all R2s of all r1 and TCY models of ECO rubber models, their chain numbers, and other information. (2) Calculate the distance between all R1 and R2; when the distance between them is within a certain distance, delete the chlorine atom connected to R1 and the hydrogen atom connected to R2, and then connect R1 and R2 to form a carbon–sulphur bond, as shown in Figure 5. When the degree of crosslinking reaches 80, a vulcanised diblock copolymer epichlorohydrin rubber model is formed. The total energy inside the model system is too high, and the internal configuration is extremely unstable. In order to make the molecular system reach the configuration with the minimum energy, it is necessary to carry out a structure optimisation (Geometry Optimization) of the model, and the method of Smart Minimization was chosen; the accuracy was chosen as Fine, the energy convergence criterion was $1\times 10^{-4}\;\mathrm{kcal}\cdot {\mathrm{mol}}^{-1}$, the maximum number of iteration steps was $5\times 10^{4}$, the external pressure was 0.0001 GPa, and when the energy change tended to the level, it was regarded that the optimised model was a reasonable configuration.

### 2.2. Construction of CNTs/ECO Models for Different Pipe Sizes

**Figure 6:**
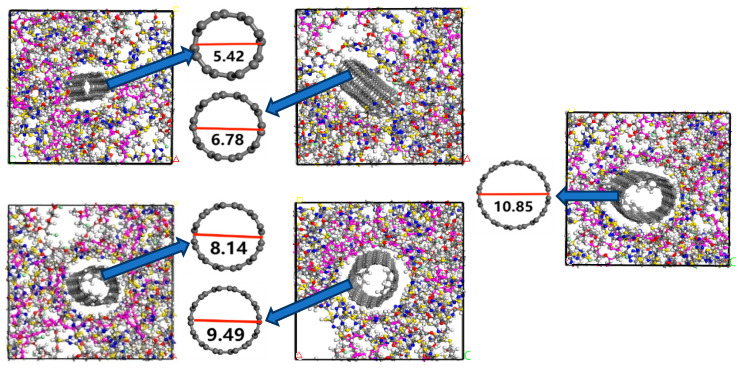
CNTs/ECO composites with different pipe diameter of CNTs.

Five CNTs with different diameters of 5.42 Å, 6.78 Å, 8.14 Å, 9.49 Å, and 10.85 Å were established using the same methodological steps. Then, the CNTs were hydrogenated and filled into the epichlorohydrin rubber with a crosslinking degree of 80 to construct five initial models of CNTs/ECO composites with different carbon nanotube diameters, and then the five initial models were subjected to geometrical optimisation, annealing, NVT, and NPT to obtain the final models, as shown in Figure 6.

## 3. Results and Discussion

### 3.1. Free Volume Fraction

Free volume is the volume of the random distribution of molecular chains in the model, in the gaps that exist between the chains [8]. The space occupied by the molecular chains throughout the model is called occupied volume. The free volume ensures that there is space for small molecules to move, so the movement of molecular chains in the model can be studied to some extent through the free volume, and the free volume fraction (FFV) is the ratio of the free volume to the total volume, and its expression is shown in Equation (1):

(1) $$FFV=\frac{V_{F}}{V_{F}+V_{O}}$$

where $V_{F}$ is the free volume of the model, and $V_{O}$ is the occupied volume of the model.

**Figure 7:**
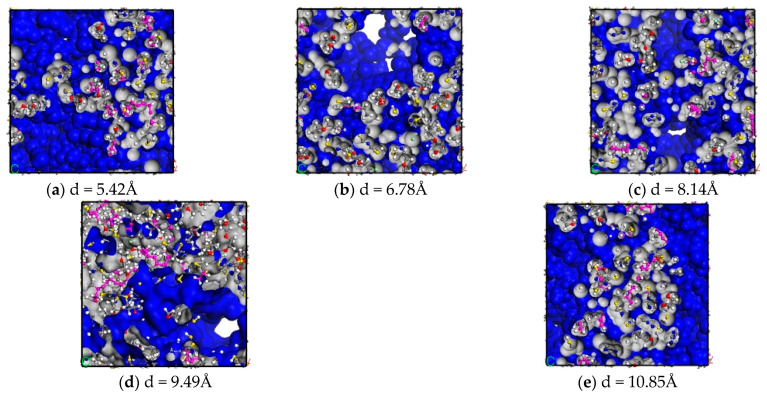
Free volume and occupied volume of CNTs/ECO composites with different diameters.

**Figure 8:**
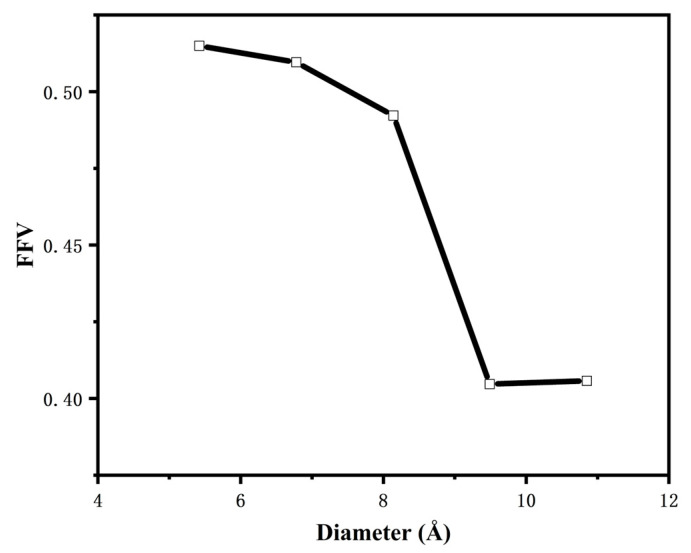
Free volume fraction of CNTs/ECO composites with different diameters.

However, because the probe molecules also have a certain volume, when the hard-sphere probe method is applied to find the free volume of the model, the probe molecules can only probe the volume space that is larger than their own volume, and they cannot probe the volume space with volume smaller than the probe molecules; however, the volume of this space is usually too small for the other mediums to enter, so the effect it produces can be ignored [9]. As shown in Figure 7, where the blue part is the free volume in the model and the grey part is the occupied volume in the model, the free volume fraction of CNTs/ECO rubber composites with different tube diameters is obtained, and the results are shown in Figure 8.

As can be seen from the figure, the free volume fractions of the composites with tube diameters of 5.42 Å, 6.78 Å, 8.14 Å, 9.49 Å, and 10.85 Å are 0.515, 0.51, 0.492, 0.404, and 0.405, respectively. The free volume fraction of CNTs/ECO composites decreases with the increase in CNT tube diameter, which is attributed to the fact that the attraction induced by the surface of CNTs increases gradually with the increase in the diameter of the CNTs, resulting in the enhancement of the intermolecular interaction force, which reduces the free volume fraction of the epichlorohydrinsole rubber, and the cold resistance property is reduced.

### 3.2. Glass Transition Temperature

**Figure 9:**
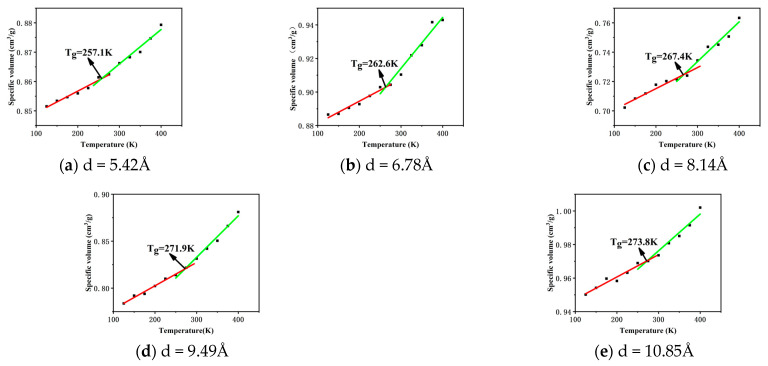
*T*_g_ of CNTs/ECO composites with different diameters.

The glass transition temperature (*T*_g_) is the temperature at which an amorphous material transforms from a glassy state to a highly elastic state [10]. The glass transition temperatures of CNTs with different tube diameters were simulated using the fitted curve of temperature versus specific volume to calculate the glass transition temperatures of CNTs with different tube diameters and to analyse the effect of the diameter of the CNTs on the crosslinked chloro ether rubbers, and the results are shown in Figure 9.

With the data shown in Figure 9, we can clearly observe the effect of CNTs with different tube diameters on the glass transition temperature of the composites. The *T*_g_ of the composites reached 257.1 K, 262.6 K, 262.6 K, 262.4 K, 267.4 K, 271.9 K, and 273.8 K when the tube diameters of CNTs were 5.42 Å, 6.78 Å, 8.14 Å, 9.49 Å, and 10.85 Å. This trend is clear for 267.4 K, 271.9 K, and 273.8 K. This trend clearly indicates that the glass transition temperature of the composites exhibits a gradual increase as the tube diameter of the CNTs increases. This increase is not coincidental but is due to the change in the interaction force between CNTs and epichlorohydrin rubber. When the length is the same as the diameter of the CNTs increases, the contact area with the epichlorohydrin rubber also increases correspondingly, and the increased contact area makes the interaction between the molecules of CNTs and epichlorohydrin rubber closer and more frequent, which strengthens the interaction force between the two, and the movement of molecular chains is subjected to greater constraints. This constraint effect makes the stiffness of the epichlorohydrin rubber increase, which improves the overall mechanical properties of the composite material, but the lower glass transition temperature represents a better cold resistance, so the cold resistance is enhanced with the reduction in the pipe diameter.

### 3.3. Mean Square Displacement

Mean square displacement (MSD) represents the sum of the squares of the difference between the absolute value of the distance r (t) and the initial position r (0) moved by microscopic particles within the system at a fixed time t, characterising the activity of the particles within the system [11], which is given by

(2) $$\mathrm{MSD}={\langle \left|r_{i}(t)-r_{i}(0)\right|\rangle }^{2}$$

**Figure 10:**
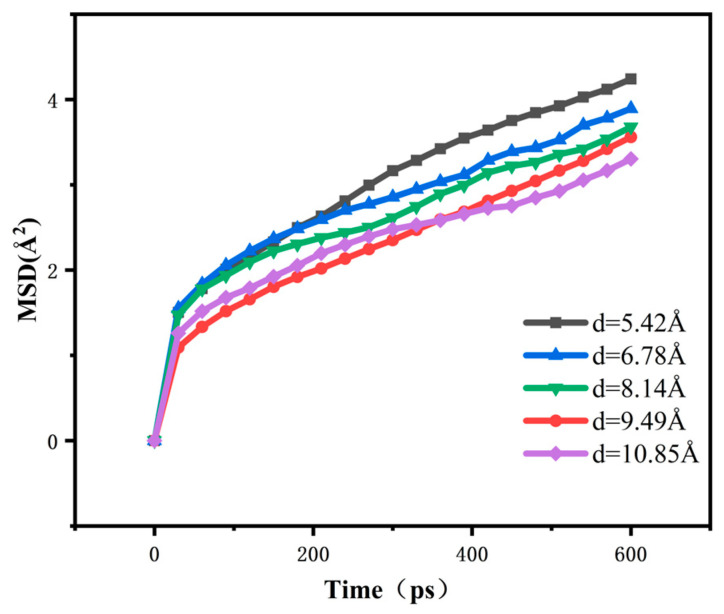
The mean square displacement of CNTs/ECO composites with different diameters.

The simulations of the mean square displacements of CNTs/ECO composites with different tube diameters were carried out, and the results are shown in Figure 10. From the figure, we can clearly observe that the trend line of the mean square displacement is the highest when the tube diameter of the CNTs reaches 5.42 Å. This indicates that at this tube diameter, the molecular chain movement inside the composite is freer and less restricted. When we see that the diameter of the tube is 10.85 Å, the trend line of the mean square displacement is the lowest, which means that as the diameter of the CNTs increases gradually, the mean square displacement of the composites shows a decreasing trend.

This phenomenon reveals that the molecular chain motility inside the composites is affected. As the diameter of CNTs increases, the interaction force between them and the rubber matrix is also enhanced. This enhanced interaction force leads to a gradual decrease in the molecular chain movement ability in the composite, i.e., the movement of molecular chains inside the material is more strongly restricted.

From a micromechanical point of view, the increase in the diameter of the CNTs leads to an increase in the contact area with the rubber matrix, which in turn strengthens the interaction between them. This enhanced interaction not only affects the molecular chain motion, but also positively influences the stability of the whole composite. As the molecular chain motion is more strongly restricted, the stability of the system is correspondingly enhanced.

### 3.4. Turning Radius

The radius of gyration, or radius of inertia, is a key physical concept in dynamics. It describes the distance from a point to the axis of rotation at the differential level, assuming that the mass of the object is concentrated at that point. Specifically, the size of the radius of gyration is obtained by dividing the object’s moment of inertia with its total mass and taking the square root. The moment of inertia, in this case, is the physical quantity that measures the amount of inertia of an object as it rotates.

In molecular dynamics simulations, the radius of gyration serves as a key indicator of the distribution of rubber molecular chains, reflecting the degree of bending of the molecular chains. Specifically, it is defined as the square root of the distance from an atom in a molecular chain to the centre of mass of the molecular chain. If the radius of gyration of a model is small, it means that the molecular chains in the model are more entangled. In this study, the radius of gyration was calculated and analysed using the Analysis function in the Forcite module in Materials Studio, and the expression of the radius of gyration in molecular dynamics is

(3) $$R_{g}^{2}=\frac{\sum_{n}^{i=1}m_{i}r_{i}^{2}}{\sum_{n}^{i=1}m_{i}}$$

where R_g_ is the radius of gyration of the molecular chain, n is the number of all atoms in the molecular chain, m_i_ is the mass of an atom, and r_i_ is the distance between the atom and the centre of mass of the molecular chain.

The values of the radius of gyration for the five models were simulated and are shown in Table 1. They reflect the effect of different carbon nanotube diameters on the molecular chain of crosslinked epichlorohydrin rubber.

As can be seen from Table 1, with the increase in the diameter of the carbon nanotube, the radius of gyration of the composite material is basically a decreasing trend; when the radius of gyration is small, it means that the molecular chains in the system are more closely filled together, which means that the material has higher structural stability and stronger mechanical properties. With the increase in tube diameter, CNTs and crosslinked epichlorohydrin rubber have stronger interaction; this strong interaction can be the better adsorption of molecular chains around the CNTs, and due to the excellent mechanical properties and stability of CNTs, when the molecular chains are adsorbed around them, the overstretching of the molecular chains is impeded, and they can better resist the tensile deformation, which improves the tensile properties of the composites.

### 3.5. Radial Distribution Function

**Figure 11:**
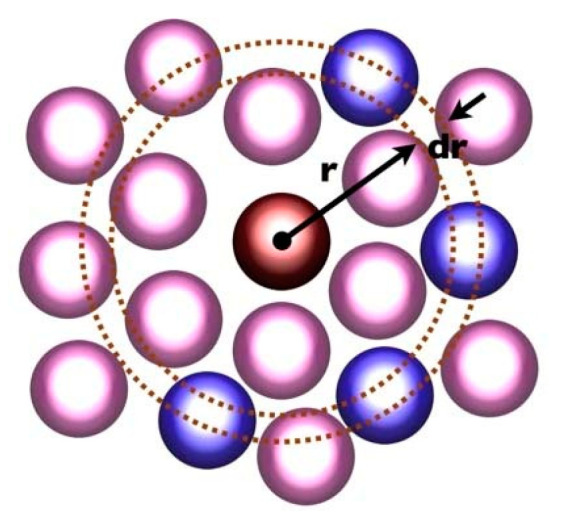
RDF diagram.

**Figure 12:**
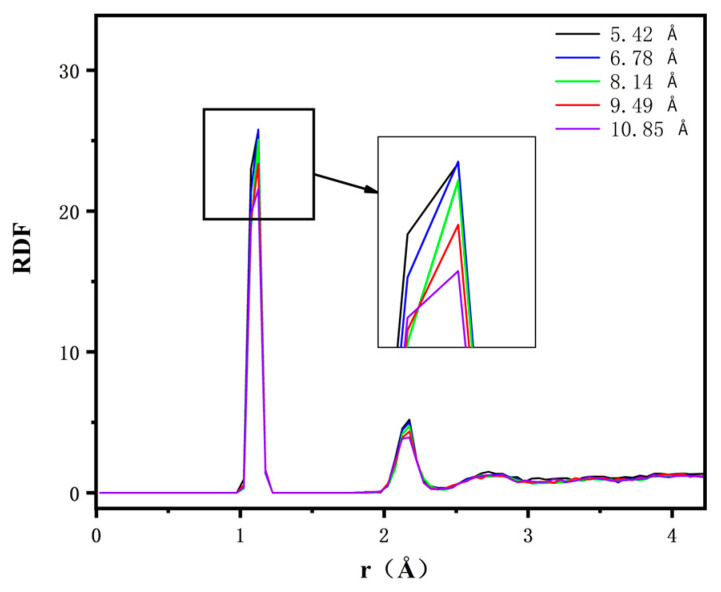
RDF of CNTs/ECO composites C and H with different diameters.

Radial distribution function (RDF) is an important physical feature that can reflect the microstructure of a material and is an important parameter for analysing the distribution of atoms in a model. RDF refers to the ratio of the densities of other atoms to the density of an atom around that atom in a spherical range of a specified radius r (shown in Figure 11). In layman’s terms, it is the number of other particles that find another atom in a spherical space of radius r, given the position of a particular microscopic particle. Its formula is expressed as Equation (4), for the atomic arrangement of the close, reasonable configuration of the system, the value of the radial distribution function will first rise rapidly to the peak, and then down to 1, gradually stabilised.

(4) $$g_{AB}\left(r\right)=\frac{n_{B}v}{4\pi r^{2}drN_{B}}$$

where $n_{B}$ is the number of B atoms around A atoms, $N_{B}$ is the number of B atoms, and v is the volume of the model. In this study, we first grouped the carbon atoms and hydrogen atoms in the model through Edit Sets in the Edit module in Materials Studio, set the carbon atom group as A and the hydrogen atom group as B, and then used the Analysis function in the Forcite module to calculate and analyse the radial distribution function of the carbon atom group and the hydrogen atom group in the model. The results are shown in Figure 12.

From the figure, it can be seen that the peaks of the RDF curves of C and H atoms for the composites with tube diameters of 5.42 Å, 6.78 Å, 8.14 Å, 9.49 Å, and 10.85 Å are 25.74, 25.7, 25.08, 23.37, and 21.58, respectively. It can be seen that the radial distribution function shows a gradual decrease with the increasing diameter of CNTs. This phenomenon arises mainly from the unique structural properties of CNTs. Their internal space is relatively large, and when the tube diameter of CNTs gradually increases, their internal space also expands, which leads to a consequent decrease in the number of hydrogen atoms found by each carbon atom, and this decrease is not due to the fact that the total number of hydrogen atoms is decreasing, but rather to the fact that with the increase in the tube diameter, the interactions between the carbon atoms and the hydrogen atoms are affected by a larger spatial distance and a more complex geometrical structure. As a result, the number of hydrogen atoms that can be found per carbon atom decreases despite the increased space inside the carbon nanotube, which in turn leads to a decrease in the radial distribution function.

### 3.6. Binding Energy

In order to further analyse the mechanism of CNTs’ influence on the performance of crosslinked epichlorohydrin rubber from the microscopic level to make use of the intermolecular binding energy, the magnitude of its value is equal to the interaction energy between molecules, as shown in Equations (5) and (6), so the binding energy to a certain extent can be expressed as the intermolecular interaction force in the system:

(5) $$E_{bind}=-E_{inter}$$

(6) $$E_{inter}=E_{Total}-E_{CNTs}-E_{ECO}$$

where $E_{bind}$ is the binding energy between CNTs and ECO, $E_{inter}$ is the interaction energy between CNTs and ECO, $E_{Total}$ is the total energy of CNTs/ECO composites, $E_{CNTs}$ is the energy of CNTs in the CNTs/ECO composites, and $E_{ECO}$ is the energy of ECO in the CNTs/ECO composites. In this study, the Energy task of the Forcite module in Materials Studio was used to calculate the energy of the CNTs, ECO, and CNTs/ECO composites, and then the intermolecular interaction energy was obtained using Equations (3)–(6), and then the binding energy in the five models was obtained using Equation. The results are shown in Table 2.

As shown in Table 2, the binding energies of the composites with carbon nanotube diameters of 5.42 Å, 6.78 Å, 8.14 Å, 9.49 Å, 10.85 Å were 284.14 Kcal/mol, 301.11 Kcal/mol, 370.92 Kcal/mol, 391.91 Kcal/mol, and 425.33 Kcal/mol, respectively. It can be seen that with the increase in the diameter of the CNTs, the contact area with the epichlorohydrinsole rubber increases, and the total bonding energy rises, which means that the interactions between the CNTs and the epichlorohydrinsole rubber are stronger, and the bonding between them is stronger. The analysis of the above simulation results is verified by the fact that the mechanical properties of the crosslinked epichlorohydrin rubber are enhanced.

## 4. Conclusions

The effect of CNTs with different tube diameters on the properties of crosslinked epichlorohydrin rubber was investigated. A model of CNTs was created using Materials Studio, and CNTs with different tube diameters were added to crosslinked epichlorohydrin rubber to analyse the effect of carbon nanotube diameters on the free volume, glass transition temperature, MSD radius of gyration, RDF, and binding energy of the crosslinked epichlorohydrin rubber at a microscopic level. The main conclusions obtained are as follows:

1. The free volume fraction and glass transition temperature of different carbon nanotube diameter composites were analysed and compared: with the increase in tube diameter, the free volume fraction of the composites decreased by 21.36%, and the *T*_g_ of the composites increased by 6.5%. The reason was that with the increase in CNT tube diameter, the contact area with epichlorohydrin rubber also increased, which made the interaction between CNTs and epichlorohydrin rubber molecules closer, enhanced the force between them, and increased the constraint of the movement of molecular chains, thus reducing the free volume fraction, increasing the stiffness of molecular chains, and increasing *T*_g_.
2. The mean square displacement and radius of gyration of different carbon nanotube diameter composites were analysed and compared. With the increase in the tube diameter, the rms displacement of the composites decreases, and the radius of gyration decreases by 3.18 Å. The reason is that the increase in the carbon nanotube diameter strengthens the molecular interactions in the system, which results in a stronger restriction of the molecular chains, and so the rms displacement and the radius of gyration of the composites both decrease.
3. The binding energy and the radial distribution function between the C and H atom groups of the five carbon nanotube diameter composites were analysed and compared, and the results show that with the increase in the tube diameter, the radial distribution function of the C and H atom groups in the system decreases gradually, and the binding energy increases gradually. The increase in the binding energy proves that the interaction force in the system is enhanced, which validates the previous results.

## Figures and Tables

**Table 1:** Radii of gyration of CNTs/ECO composites with different diameters.

CNTs Pipe Diameter (Å)	R_g_ (Å)
5.42	18.43
6.78	18.88
8.14	18.39
9.49	17.43
10.85	15.25

**Table 2:** Four energy values of CNTs/ECO composites with different aspect ratios of CNTs.

CNT Tube Diameter (Å)	$E_{bind}$ (Kcal/mol)	$E_{Total}$ (Kcal/mol)	$E_{CNTs}$ (Kcal/mol)	$E_{ECO}\;(\mathrm{Kcal}/\mathrm{mol})$
5.42	284.14	74.75	11,165.59	−10,806.70
6.78	301.11	3051.43	13,764.73	−10,412.20
8.14	370.92	5381.51	16,472.72	−10,720.29
9.49	391.91	9723.28	19,146.15	−9030.96
10.85	425.33	11,893.71	21,805.09	−9486.05

## Data Availability

The original contributions presented in this study are included in the article/Supplementary Materials; further inquiries can be directed to the corresponding author.

## Supplementary Materials

The following supporting information can be downloaded at: https://www.mdpi.com/article/10.3390/polym16172419/s1, Table S1. Detailed data on the free volume fraction of crosslinked CNTs/ECO for different carbon nanotube diameters. Table S2. Detailed Tg data for crosslinked CNTs/ECO with carbon nanotube diameter of 5.42 Å. Table S3. Detailed Tg data for crosslinked CNTs/ECO with carbon nanotube diameter of 6.78 Å. Table S4. Detailed Tg data for crosslinked CNTs/ECO with carbon nanotube diameter of 8.14 Å. Table S5. Detailed Tg data for crosslinked CNTs/ECO with carbon nanotube diameter of 9.49 Å. Table S6. Detailed Tg data for crosslinked CNTs/ECO with carbon nanotube diameter of 10.85 Å. Table S7. Detailed MSD data of crosslinked CNTs/ECO with different carbon nanotube diameters. Table S8. Detailed RDF data for crosslinked CNTs/ECO with different carbon nanotube diameters.
