# Supplementary material for: Molecular Dynamics Simulation of Effect of Carbon Nanotube Diameter on Properties of Crosslinked Epichlorohydrin Rubbers
Source: Polymers (Basel). 2024 Aug 26;16(17):2419. doi: 10.3390/polym16172419 (PMC11397257; doi:10.3390/polym16172419)
Supplement: Supplementary file 1 [file polymers-16-02419-s001.zip › polymers-3096393-supplementary.pdf]

Table S1. Detailed data on the free volume fraction of crosslinked CNTs / ECO for different carbon nanotube diameters.

Diameter（Å）	FFV
5.42	0.51496
6.78	0.5096
8.14	0.49219
9.49	0.40468
10.85	0.40573

Table S2. Detailed T_g data for crosslinked CNTs / ECO with carbon nanotube diameter of 5.42 Å.

Temperature(K)	Specific volume(cm^3/g)
125	0.95016
150	0.95417
175	0.95967
200	0.9583
225	0.96318
250	0.96895
275	0.97016
300	0.97357
325	0.9808
350	0.98505
375	0.99147
400	1.00203

Table S3. Detailed T_g data for crosslinked CNTs / ECO with carbon nanotube diameter of 6.78 Å.

Temperature(K)	Specific volume(cm^3/g)
125	0.85152
150	0.85341
175	0.85468
200	0.85597
225	0.85775
250	0.86142
275	0.86242
300	0.86624
325	0.86827
350	0.87004
375	0.87468
400	0.87932

Table S4. Detailed T_g data for crosslinked CNTs / ECO with carbon nanotube diameter of 8.14 Å.

Temperature(K)	Specific volume(cm^3/g)
125	0.88656
150	0.887
175	0.89057
200	0.89278
225	0.89765
250	0.90289
275	0.9043
300	0.91035
325	0.92184
350	0.92791
375	0.94161
400	0.94293

Table S5. Detailed T_g data for crosslinked CNTs / ECO with carbon nanotube diameter of 9.49 Å.

Temperature(K)	Specific volume(cm^3/g)
125	0.70223
150	0.70835
175	0.71178
200	0.71784
225	0.72028
250	0.72154
275	0.72403
300	0.73436
325	0.74364
350	0.74507
375	0.75065
400	0.76332

Table S6. Detailed T_g data for crosslinked CNTs / ECO with carbon nanotube diameter of 10.85 Å.

Temperature(K)	Specific volume(cm^3/g)
125	0.78372
150	0.79197
175	0.79392
200	0.80225
225	0.81
250	0.81392
275	0.82175
300	0.83147
325	0.84207

350	0.85043
375	0.86617
400	0.88102

Table S7. Detailed MSD data of crosslinked CNTs / ECO with different carbon nanotube diameters.

Time(ps)	MSD(Å^2)				
	d=5.42Å	d=6.78 Å	d=8.14 Å	d=9.49 Å	d=10.85 Å
0	0	0	0	0	0
30	1.50358	1.09163	1.54967	1.47441	1.25961
60	1.78221	1.33496	1.83473	1.7797	1.5172
90	2.00916	1.51818	2.05546	1.9375	1.67699
120	2.16523	1.65895	2.22491	2.09482	1.78814
150	2.33012	1.80466	2.36794	2.22479	1.92429
180	2.49827	1.9197	2.48596	2.30525	2.05256
210	2.63409	2.01855	2.59214	2.38061	2.19687
240	2.81151	2.13395	2.70069	2.44016	2.2974
270	2.99491	2.24648	2.77363	2.50173	2.39586
300	3.16416	2.35106	2.85616	2.61264	2.47873
330	3.28612	2.47251	2.94703	2.74531	2.52913
360	3.42253	2.59246	3.03811	2.89388	2.5842
390	3.54682	2.68448	3.11729	2.99436	2.65756
420	3.63967	2.8114	3.28879	3.14186	2.72658
450	3.75364	2.92831	3.38961	3.22074	2.75355
480	3.84541	3.04382	3.43548	3.26516	2.84857
510	3.92588	3.16584	3.52686	3.35784	2.92696
540	4.02814	3.28266	3.69993	3.42018	3.0548
570	4.1204	3.42051	3.78243	3.53658	3.16593
600	4.24113	3.56095	3.89321	3.67731	3.30474

Table S8. Detailed RDF data for crosslinked CNTs / ECO with different carbon nanotube diameters.

r(Å)	RDF				
	d=5.42Å	d=6.78 Å	d=8.14 Å	d=9.49 Å	d=10.85 Å
0.975	0	0	0	0	0
1.025	0.96133	0.45789	0.32077	0.57228	0.39916
1.075	22.99704	21.3352	18.80985	19.29805	19.77817
1.125	25.73702	25.8007	25.07503	23.36519	21.58023
1.175	1.64606	1.39385	1.58669	1.62327	1.51885
1.225	0	0	0	0	0
1.275	0	0	0	0	0
1.325	0	0	0	0	0
1.375	0	0	0	0	0
1.425	0	0	0	0	0

1.475	0	0	0	0	0
1.525	0	0	0	0	0
1.575	0	0	0	0	0
1.625	0	0	0	0	0
1.675	0	0	0	0	0
1.725	0	0	0	0	0
1.775	0	0	0	0	0
1.825	0	0	0	0.01641	0.03148
1.875	0.01796	0.06843	0.03196	0.0311	0
1.925	0.01704	0.04869	0.03032	0.07376	0.05659
1.975	0.09711	0.01542	0.10081	0.05606	0.09409
2.025	0.53887	0.61601	0.45208	0.65324	0.48585
2.075	2.3901	1.96956	1.69612	2.01878	2.26491
2.125	4.5579	4.44853	4.17995	3.91033	3.83152
2.175	5.2049	5.00918	4.73811	4.35665	3.93446
2.225	2.33377	2.3447	2.20137	2.31894	2.22401
2.275	0.85389	0.72048	1.00942	0.71825	0.69898
2.325	0.46718	0.42279	0.43647	0.32362	0.27158
2.375	0.34698	0.36253	0.24898	0.2423	0.23238
2.425	0.34355	0.23523	0.21971	0.33467	0.31205
2.475	0.4638	0.50074	0.36683	0.52654	0.36804
2.525	0.71299	0.66978	0.70489	0.68596	0.71545
2.575	0.99978	0.97056	0.87265	0.81624	0.86189
2.625	1.11782	1.15215	1.0843	1.13452	1.06525
